# A comprehensive characterisation of phaeochromocytoma and paraganglioma tumours through histone protein profiling, DNA methylation and transcriptomic analysis genome wide

**DOI:** 10.1186/s13148-023-01598-3

**Published:** 2023-12-20

**Authors:** Prodromos Chatzikyriakou, Dimitria Brempou, Mark Quinn, Lauren Fishbein, Roberta Noberini, Ioannis N. Anastopoulos, Nicola Tufton, Eugenie S. Lim, Rupert Obholzer, Johnathan G. Hubbard, Mufaddal Moonim, Tiziana Bonaldi, Katherine L. Nathanson, Louise Izatt, Rebecca J. Oakey

**Affiliations:** 1 Department of Medical and Molecular Genetics, King’s College London, London, SE1 9RT UK; 2 Division of Translational Medicine and Human Genetics, Department of Medicine, Perelman School of Medicine, University of Pennsylvania, Philadelphia, PA USA; 3 Abramson Cancer Center, Perelman School of Medicine, Philadelphia, PA USA; 4 Division of Endocrinology, Diabetes and Metabolism in the Department of Medicine Perelman School of Medicine, University of Pennsylvania, Philadelphia, PA USA; 5 Department of Experimental Oncology, IEO, European Institute of Oncology IRCCS, Via Adamello 16, 20139 Milan, Italy; 6 Department of Biomolecular Engineering, UC Santa Cruz Genomics Institute, University of California, Santa Cruz, Santa Cruz, CA 95064 USA; 7 Department of Endocrinology, St. Bartholomew’s Hospital, Barts Health NHS Trust, and William Harvey Research Institute, Queen Mary University of London, London, UK; 8 Department of ENT and Skull Base Surgery, Guy’s and St Thomas’ NHS Foundation Trust, London, SE1 9RT UK; 9 Department of Endocrine Surgery, Guy’s and St Thomas’ NHS Foundation Trust, London, SE1 9RT UK; 10 Department of Cellular Pathology, Guy’s and St Thomas’ NHS Foundation Trust, London, SE1 9RT UK; 11 Department of Oncology and Hematology-Oncology, University of Milano, Via Festa del Perdono 7, 20122 Milan, Italy; 12 Department of Clinical Genetics, Guy’s and St Thomas’ NHS Foundation Trust, London, SE1 9RT UK; 13 Present Address: Comprehensive Cancer Centre, King’s College London, London, SE5 8AF UK; 14 Present Address: Division of Endocrinology, Metabolism and Diabetes, Department of Medicine, University of Colorado School of Medicine, Aurora, CO USA; 15 Present Address: Imperial College Healthcare NHS Trust, London, UK

**Keywords:** Phaeochromocytoma, Paraganglioma, DNA methylation, Epigenetics, Succinate dehydrogenase, Histone post-translational modifications, Mass spectrometry, SDH, Cancer predisposition, Germline pathogenic variant

## Abstract

**Background:**

Phaeochromocytomas and paragangliomas (PPGLs) are rare neuroendocrine tumours. Pathogenic variants have been identified in more than 15 susceptibility genes; associated tumours are grouped into three Clusters, reinforced by their transcriptional profiles. Cluster 1A PPGLs have pathogenic variants affecting enzymes of the tricarboxylic acid cycle, including succinate dehydrogenase. Within inherited PPGLs, these are the most common. PPGL tumours are known to undergo epigenetic reprograming, and here, we report on global histone post-translational modifications and DNA methylation levels, alongside clinical phenotypes.

**Results:**

Out of the 25 histone post-translational modifications examined, Cluster 1A PPGLs were distinguished from other tumours by a decrease in hyper-acetylated peptides and an increase in H3K4me2. DNA methylation was compared between tumours from individuals who developed metastatic disease versus those that did not. The majority of differentially methylated sites identified tended to be completely methylated or unmethylated in non-metastatic tumours, with low inter-sample variance. Metastatic tumours by contrast consistently had an intermediate DNA methylation state, including the ephrin receptor *EPHA4* and its ligand *EFNA3*. Gene expression analyses performed to identify genes involved in metastatic tumour behaviour pin-pointed a number of genes previously described as mis-regulated in Cluster 1A tumours, as well as highlighting the tumour suppressor *RGS22* and the pituitary tumour-transforming gene *PTTG1*.

**Conclusions:**

Combined transcriptomic and DNA methylation analyses revealed aberrant pathways, including ones that could be implicated in metastatic phenotypes and, for the first time, we report a decrease in hyper-acetylated histone marks in Cluster 1 PPGLs.

**Supplementary Information:**

The online version contains supplementary material available at 10.1186/s13148-023-01598-3.

## Introduction

Phaeochromocytomas (PCCs) and paragangliomas (PGLs), collectively referred to as PPGLs, are rare neuroendocrine tumours derived from chromaffin cells in the adrenal medulla and the sympathetic and parasympathetic paraganglia, respectively. PPGLs have the highest degree of heritability of any cancer type [1], and despite their relative rarity in the general population, 25% of PPGLs are life-threatening neoplasms [2]. Standard treatment of PPGLs is total surgical resection, when possible. No treatments are highly effective for metastatic PPGLs [3]; options include radiopharmaceuticals [4], chemotherapy [5] and external-beam radiotherapy [6]. Understanding more about the mechanisms of tumour formation is therefore a priority for the development of treatment regimens and disease screening stratification.

Around 40% of PPGLs are caused by inherited germline pathogenic variants in over 15 susceptibility genes that can be readily tested for [7]. It is estimated that an additional 30% of PPGLs arise due to somatic pathogenic variants. Somatic variants may arise in the same susceptibility genes as in the germline. There are also a number of exclusively somatic variants implicated in PPGL development such as *EPAS1, HRAS, BRAF, CSDE1* and *UBTF-MAML3* fusion [8]. PPGLs are grouped in clusters based on their transcriptional profile [9] and tumorigenic mechanism underlying their aetiology [10].

Cluster 1A tumours have pathogenic variants in enzymes of the tricarboxylic acid (TCA) cycle, including in the genes encoding the different subunits of succinate dehydrogenase *SDHA*, *SDHB*, *SDHC*, *SDHD* and the assembly factor *SDHAF2* (collectively *SDHx*), fumarate hydratase *FH*, isocitrate dehydrogenase *IDH1*/*2*, malate dehydrogenase 2 *MDH2,* glutamic-oxaloacetic transaminase 2 *GOT2* and, rarely, the also metabolism-related *SCL25A11* and *SUCLG2* [11]. Cluster 1B tumours harbour pathogenic variants in genes involved in oxygen sensing (*VHL*, *EPAS1*, *EGLN1/2*). Cluster 2 tumours are characterised by aberrant kinase signalling due to variants in genes such as *NF1*, *RET, HRAS, MAX* and *TMEM127*. Recently, somatic variants in *CSDE1* and in-frame RNA fusion transcripts of the *UBTF-MAML3* genes that activate Hedgehog and Wnt pathways have been identified, comprising Cluster 3 [1].

Gene expression is modulated by epigenetic mechanisms in normal development with DNA methylation and post-translational modifications (PTMs) of histone proteins mediating this [12]. Epigenetic reprogramming occurs in normal development [13], but in tumour cells, genome-wide reprogramming has been associated with tumour development [14], while tumour suppressor genes are often silenced via DNA hypermethylation of their promoters despite more global hypomethylation [15]. In contrast, Cluster 1A PPGL tumours undergo dramatic epigenetic reprograming, including genome-wide DNA hypermethylation and alterations to histones [16], which have been associated with gene expression dysregulation [9, 17]. These epigenetic changes have been associated with epithelial-to-mesenchymal transition, which is involved in tumour invasion and aggressiveness [18, 19], impaired DNA damage repair [20] and invasive behaviour [21].

*SDHB* tumours (Cluster 1A) have an increased risk of developing distant metastases [22, 23]. Tumours with pathogenic variants in Cluster 1A genes are characterised by the accumulation of oncometabolites [24–27], which contribute to tumorigenesis [28] and their accumulation has been linked to epigenetic aberrations [29]. Dysregulation of DNA methylation in PPGL Cluster 1A tumours identified the CpG island methylator phenotype (CIMP) as a prognostic factor associated with malignant behaviour [30].

Using early DNA methylation technologies, the first such study assayed a small number (1536) of cytosines in 29 PPGLs [31]. The development of higher resolution approaches provided more coverage genome wide including one study that characterised four adrenal samples and 39 PPGL tumours, but only one with a Cluster 1A tumour [32] and another study involving 19 PPGL tumours, again with only one tumour harbouring a Cluster 1A variant [33]. The PPGL arm of The Cancer Genome Atlas (TCGA) network undertook a comprehensive molecular characterisation of PPGL tumours using whole genome sequencing, transcriptomics and DNA methylation arrays for 173 cases of which 15 were *SDHB* and three were *SDHD* tumours [1]. Within this cohort, eight (5%) of the tumours were metastatic, with an additional nine (5%) exhibiting aggressive disease as defined by local recurrence or loco-regional disease. More recently, a study analysed PPGLs with single-nuclei and bulk RNA-seq to expand on the molecular classification of these tumours and describe their microenvironment [34]. Here, we explore the hypothesis of whether Cluster 1A PPGL tumours undergo distinct epigenetic changes involving both altered histone modifications and DNA hypermethylation compared to other PPGL subtypes and how these could be linked to clinically-relevant phenotypes.

## Materials and methods

### Patient cohort

Herein, we report on global histone PTMs, DNA methylation and the transcriptomes of PPGL tumours of known genetic aetiology, along with their clinical phenotypes, providing a comprehensive transcriptomic and epigenetic dataset of PPGLs. Histone PTMs of 30 PPGL tumours were examined by mass spectrometry (MS) DNA methylation profiles of 52 PPGL tumours, and two normal adrenal medulla samples were performed. The transcriptomes of 29 PPGL tumours, with phenotype information, were generated (INA, KLN). DNA methylation analysis was performed on 28 of these same samples. This allows for comparisons to be made between gene expression and DNA methylation in the same individuals (Table 1). Tumour samples were obtained from several sources (Additional file 1: Figure S1):

- RNA sequencing data were obtained from 29 PPGL samples provided by the University of Pennsylvania (UPenn) (INA, KJN)
  - DNA methylation profiles were analysed on 52 samples in total obtained from (Table 1): Sixteen PPGL tumours from the public repository, Gene Expression Omnibus (GEO accession GSE111336 [35]). One sample (PPGL 11) did not pass quality control and was excluded from subsequent analyses.
  - Twenty-eight PPGL tumours from the UPenn cohort (LF, INA, KLN)
  - Six tumour samples from Guy’s & St. Thomas’ NHSFT (LI).
  - Two normal adrenal medulla samples from Barts Health NHSFT (NT) isolated from tumour-free regions from two individuals with *RET* PCC (ESL) were analysed. These samples were preserved in FFPE and isolated using laser-capture microdissection (ZEISS PALM CombiSystem) from areas that were clear of disease, as delineated by a specialist endocrine histopathologist (MM).

**Table 1:** Tumour samples are listed along with their pathogenic variant detected either by diagnostic test or by whole genome sequencing where known (column 2)

	(ALL) *N* = 52	*N*
Group		52
*HRAS*	2 (3.85%)	
*IDH3B*	1 (1.92%)	
*NF1*	1 (1.92%)	
Normal	2 (3.85%)	
*RET*	1 (1.92%)	
*SDHA*	1 (1.92%)	
*SDHB*	14 (26.9%)	
*SDHD*	5 (9.62%)	
Sporadic	20 (38.5%)	
*VHL*	5 (9.62%)	
Location		50
EAPGL	21 (42.0%)	
HNPGL	7 (14.0%)	
PCC	22 (44.0%)	
Metastatic		50
No	20 (40.0%)	
Unknown	22 (44.0%)	
Yes	8 (16.0%)	
Primary		50
No	4 (8.00%)	
Unknown	22 (44.0%)	
Yes	24 (48.0%)	
Recurring		50
No	21 (42.0%)	
Unknown	22 (44.0%)	
Yes	7 (14.0%)	
Clinically aggressive		50
No	17 (34.0%)	
Unknown	22 (44.0%)	
Yes	11 (22.0%)	
ATRX double mutation:		50
No	24 (48.0%)	
Unknown	22 (44.0%)	
Yes	4 (8.00%)	
Cluster		52
Cluster 1A	21 (40.4%)	
Cluster 1B	5 (9.62%)	
Cluster 2	4 (7.69%)	
Normal	2 (3.85%)	
Sporadic	20 (38.5%)	
Sex		52
F	15 (28.8%)	
M	21 (40.4%)	
Unknown	16 (30.8%)	
Tissue		52
Normal adrenal medulla	2 (3.85%)	
Tumour	50 (96.2%)	

The location of the tumour site is listed (location) since this is relevant to DNA methylation pattern. The behaviour of the tumour is described as aggressive or metastatic or neither. EAPGL: Extra-adrenal paraganglioma (i.e. of the thorax, abdomen or pelvis). HNPGL: Head and neck paraganglioma. PCC: Phaeochromocytoma

Pathology tissue analysis of histones for mass spectrometry (PAT-H-MS) was performed on 31 samples (1 normal adrenal and 30 PPGL samples) collected from GSTTFT (Additional file 7: Table S1) [35].

For 28 tumours, detailed clinical data were available; eight (28.6%) had been classified as metastatic and 20 (71.4%) as non-metastatic. Twenty-four (85.7%) were primary tumours and four (14.3%) were from distant metastases. Seven tumours were recurring (25%) and 11 (39.3%) tumours were classified as clinically aggressive (Table 1 and Additional file 1: Figure S1). Metastatic disease was classified as the occurrence of metastases in non-chromaffin tissues including lymph nodes. Clinically aggressive disease events were defined by the occurrence of distant metastases, positive regional lymph nodes, or local recurrence [1]. The average follow-up time was 77 months (range 2–194 months). Overall, 21 (40.4%) samples belonged to Cluster 1A (*SDHA*, *SDHB*, *SDHD*, *IDH3B*), 5 (9.6%) to Cluster 1B (*VHL*), 4 in Cluster 2 (*RET*, *NF1*, *HRAS*) and 20 (38.5%) had no identified germline pathogenic variants in the PPGL predisposing genes (sporadic tumours) (Table 1).

### DNA methylation analysis

Tumour DNA was extracted from ~ 20 mg fresh frozen samples using the Quick-DNA Miniprep kit (Cat. #D3024, Zymo Research, Irvine, CA, USA) according to manufacturer’s instructions. DNA was extracted from four serial FFPE-preserved sections using the Quick-DNA FFPE Miniprep kit (Cat. #D3067, Zymo Research).

Bisulphite conversion of 500 ng of genomic DNA was performed with the Zymo EZ DNA Methylation Kit (Cat. #D5001, Zymo Research) according to manufacturer’s instructions for DNA methylation analysis.

Illumina’s Infinium MethylationEPIC™ BeadChips (EPIC) assayed the DNA methylation status of ~ 850,000 CpG sites genome wide including at promoters, and other functionally relevant regions of the genome, such as enhancer elements defined by the FANTOM5 study [36], data from DNaseI hypersensitive sites revealing regions of open chromatin, enhancers identified from ENCODE [37] and miRNA promoter regions [38]. The Infinium MethylationEPIC™ Kit (Cat. # WG-317-1001, Illumina) was used according to manufacturer’s instructions for eight samples of 400 ng of bisulphite-converted DNA per array and analysed using the Illumina iScan. DNA samples (KLN) were analysed at the GSTT BRC Genomics Core Facility. Eight DNA samples were assayed by the Human Genomics Facility of Erasmus University Medical Centre, Netherlands (Additional file 7: Table S1).

All data were analysed according to principles of best practice [39–43]. DNA methylation was measured at each CpG site by the respective probes present on the array. DNA methylation was quantified by the *β*-value = *M*/(*M* + *U* + *a*), where *M* > *0* and *U* > *0* denote the methylated and unmethylated signal intensities, respectively. Between-array, dye-bias correction was performed with the out-of-band background correction method [44] as implemented by the methylumi package (vers. 2.34.0, [45]). Intra-array normalisation of type II probes was performed with the use of Beta Mixture Quantile dilation (BMIQ) algorithm [46] using the wateRmelon package (vers. 1.32.0, [47]).

Differential DNA methylation analysis was conducted with the RnBeads package (vers. 2.4.0, [48]). Statistical significance for differential methylation was set at 5% false discovery rate (FDR) after multiple testing correction with the Benjamini–Hochberg procedure [49] and Δ*β* ≥ 0.15.

The variance of *β*-values of all filtered probes was calculated across all 52 samples, and the probes were ranked in an order of decreasing variance. Hierarchical clustering of samples was performed with pheatmap package (vers. 1.0.12) based on the 5,000 most variable probes using Manhattan distances for column aggregation and Minkowski distances for rows, with complete linkage agglomeration method.

Principal component analysis (PCA) was performed on the 50,000 most variable probes using the stats package (vers. 3.6). Ellipses representing 95% confidence intervals incorporating unobserved population parameters such as the true population mean from the bivariate distribution were added to plots.

*Β*-value densities were calculated for probes, promoter and gene bodies to analyse their frequency distributions for each methylation level. Additionally, methylation levels were plotted across promoter and gene bodies, after normalisation, to account for differences in gene length.

#### Gene expression analysis.

RNA was extracted using Trizol™. Paired-end, non-stranded libraries were prepared after poly-A tail selection using the TruSeq™ Stranded Total RNA LT Sample Prep Kit and sequenced on an Illumina HiSeq2500 with an average of 50 million reads per sample. Reads were pseudo-aligned with kallisto (vers. 0.46.1, [50]) on ENSEMBL 98 transcriptome [51] setting *k*-mer length of 31 for index construction, number of bootstraps set to 100 and enabling bias correction.

Differential gene expression analysis was performed using sleuth (vers. 0.30.0, [52]) by aggregating transcript *p* values with the Lancaster method as described Yi et al. [53]. Genes were considered differentially expressed when the effect size is ≥ 1 and statistical significance for multiple testing correction FDR < 0.05.

#### Sample preparation for histone post-translational modifications analysis by MS

Tumour tissues were transferred to the bottom of tubes through centrifugation and histones were enriched as previously described [54]. Briefly, samples were homogenised in 1 ml of phosphate-buffered saline (PBS) containing 0.1% Triton X-100 and protease inhibitors and filtered through a 100 µm cell strainer. Nuclei were isolated through a 10 min centrifugation at 2300×*g*, resuspended in the same buffer containing 0.1% sodium dodecyl sulphate (SDS), and incubated for 5 min at 37°C in the presence of 250 U of benzonase (Merck). The yield of histones was estimated by SDS-PAGE gel by comparison with known amounts of recombinant histone H3.1 (NEB), following protein detection with colloidal Coomassie staining. Approximately 2–4 μg of histones were mixed with a heavy-labelled histone super-SILAC mix, which was generated as previously described and used as an internal standard for relative quantification among multiple samples [55, 56]. Proteins from each sample were separated on a 17% SDS-PAGE gel. A band corresponding to the molecular weight of the histone octamer (H3, H4, H2A, H2B) was excised from the gel, subjected to chemical acylation with propionic anhydride and in-gel digested with trypsin. After elution from the gel, the digested peptides were subjected to an additional chemical derivatisation step of the released N-termini with phenyl isocyanate, as described [57].

#### MS analysis of histone PTMs

Peptide mixtures were separated by reversed-phase nano-liquid chromatography on an EASY-Spray column (Thermo Fisher Scientific), 25-cm long (inner diameter 75 µm, PepMap C18, 2 µm particles), which was connected online to a Q-Exactive HF instrument through an EASY-Spray™ Ion Source (Thermo Fisher Scientific). Solvent A was 0.1% formic acid (FA) in ddH2O, and solvent B was 80% acetonitrile plus 0.1% FA. Peptides were injected in an aqueous 1% TFA solution at a flow rate of 500 nl/min and were separated with a 50-min linear gradient of 10–45% B. The Q-Exactive instruments were operated in the data-dependent acquisition (DDA) mode. Survey full scan MS spectra (m/z 300–1350) were analysed in the Orbitrap detector with a resolution of 60,000–70,000 at m/z 200. The 10 most intense peptide ions with charge states comprised between 2 and 4 were sequentially isolated to a target value for MS1 of 3 × 10^6^ and fragmented by HCD with a normalised collision energy setting of 28%. The maximum allowed ion accumulation times were 20 ms for full scans and 80 ms for MS/MS, and the target value for MS/MS was set to 1 × 10^5^. The dynamic exclusion time was set to 10 s, and the standard mass spectrometric conditions for all experiments were as follows: spray voltage of 1.8 kV, no sheath and auxiliary gas flow. The mass spectrometry proteomics data have been deposited to the ProteomeXchange Consortium [58] via the PRIDE partner repository with dataset identifier PXD025689.

The acquired RAW data were analysed using Epiprofile 2.0 [59], selecting the SILAC option, followed by manual validation. For each histone modified peptide, the percentage relative abundance (%RA) was estimated by dividing the area under the curve (AUC) of each modified peptide for the sum of the areas corresponding to all the observed forms of that peptide and multiplying by 100. Light/heavy (L/H) ratios of %RA were then calculated. The AUC values for all the samples analysed are reported in Additional file 8: Table S2. Data display was carried out using Perseus [60] and GraphPad Prism (Graphpad). Statistical testing was performed using GraphPad Prism. Changes in single modified peptides between Cluster 1A samples and other samples were evaluated by multiple *t* test. Normalised L/H ratios, defined as L/H ratios of relative abundances normalised over the average value across the samples, were visualised and clustered with correlation distance and average linkage as parameters.

#### Gene set enrichment analysis of DNA methylation and transcriptomic data

To understand the biological processes related to the changes in DNA methylation data, over-representation analysis (ORA) and gene set enrichment analysis (GSEA) [61] methylGSA package (vers. 1.6.1, [62]) was used implementing the robust rank aggregation method to adjust for number of CpGs per gene [63]. The Kyoto Encyclopaedia of Genes and Genomes (KEGG) database [64] as implemented by the KEGG.db package (vers. 3.24), the Reactome database [65] as implemented by reactome.db package (vers. 1.70.0) and gene ontology (GO) terms [66] from the GO.db package (vers. 3.11.4) were used.

For gene expression analyses, functional enrichment with GSEA and ORA was performed on differentially expressed genes using WebGestalt software (vers. 2019, [67]). Parameters used were minimum number of genes for a category = 5, maximum number of genes for a category = 2000 and multiple testing adjustment with the Benjamini–Hochberg procedure.

#### Correlations between DNA methylation and RNA expression

For the 28 PPGL samples that had both DNA methylation and transcriptomic data available, the correlations between promoter methylation status and gene expression were calculated to identify candidate loci where DNA methylation changes could have affected gene expression. The levels of differential DNA methylation at promoter regions (Δ*β*) were plotted against the degree of differential expression (*b*) for the genes that had both significantly differentially methylated promoters and significantly different expression (FDR ≤ 0.05). To minimise the risk of uneven coverage or averages that are not reflective of the DNA methylation levels of promoters with too few probes in the EPIC 850k array, only promoters with more than 11 probes passing the filtering criteria were included. Pearson’s correlation coefficient *ρ* was calculated, and a linear regression model with standard error was fitted to the data using ggpubr package (vers. 0.4.0). Locally estimated scatterplot smoothing (LOESS) was used to fit the nonparametric linear model, and the curves with 95% confidence intervals were plotted using ggplot2 package (vers. 3.3.2, [68]).

#### Software

Analyses were performed in R (vers. 3.4.3, [69]), Bioconductor (vers. 3.9, [70]).

## Results

The distribution of DNA methylation both around defined regulatory features and agnostic to functional annotation was examined. PPGLs with different underlying genetic aetiologies were included to identify genotype-specific and shared features. DNA methylation data were compared to corresponding transcriptomes in the same individuals for a subset of tumour samples to interrogate whether gene expression could be linked to a mechanistic aetiology through DNA methylation. Further, the phenotypes of tumours were compared, to identify genes associated with aggressive tumour behaviour and metastasis.

### Cluster 1A tumours exhibit genome-wide alterations in DNA methylation

**Fig. 1:**
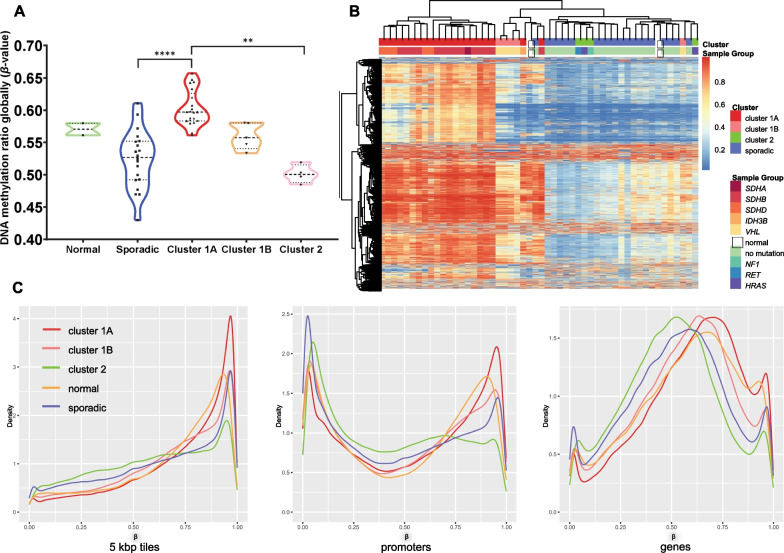
**A** Average DNA methylation levels from all the probes of the 850 K array: Cluster 1A tumours are significantly more methylated than sporadic (*p* < 0.0001, Kruskal–Wallis test) and Cluster 2 (*p* = 0.0014) tumours. In Cluster 1A, *SDHB* samples are shown as triangle and all the other samples with a star symbol. **B** Unsupervised hierarchical clustering of all samples based on the DNA methylation levels of the 5000 most variable probes. Three distinct groups are observed, largely coinciding with the clusters described in the literature previously. The *SDHx* tumours form a separate cluster with the *VHL* tumour, while sporadic and Cluster 2 tumours form one uniform group. **C** Comparison of *β*-value densities across samples from each group. Cluster 1A tumours have a higher peak on more methylated regions when the genome is divided in 5 Kbp-tiles agnostic of content and in both promoter and gene bodies

The DNA methylation profiles of 50 PPGLs (21 Cluster 1A, five Cluster 1B, four Cluster 2 and 20 sporadic tumours) plus two normal adrenal medulla samples were assayed. Global DNA methylation calculated as a methylation index (MI) [32] separates Cluster 1A and 1B tumours from sporadic, Cluster 2 tumours and normal medulla (Fig. 1A). Within Cluster 1A, *SDHB* tumours were the most hypermethylated (MI: 0.61 vs 0.58, Welch’s *t* test *p* = 0.06).

Principal component analysis (PCA) (Additional file 2: Figure S2) separated Cluster 1B from sporadic and Cluster 2 tumours confirming that much of the DNA methylation variance is driven by the pathogenic variant underlying the tumour. Unsupervised hierarchical clustering of the 5000 most variable probes segregated the tumours of Cluster 1A from the other samples (Fig. 1B), consistent with the MI values, and reflecting epigenetic changes known to occur in PPGL tumours.

The genome was divided into 5kbp serial “windows” (tiles) agnostic to content or functional relevance [48], and Cluster 1A tumours had had near complete DNA methylation across these regions. Cluster 1A samples also had the highest degree of promoter DNA methylation, a functionally relevant category with known regulatory relationship [71]. Gene bodies showed similar trends across samples with Cluster 1A tumours exhibiting a shift towards higher values of DNA methylation (Fig. 1C).

### Differential DNA methylation analysis delineates the group of hypermethylated Cluster 1A tumours

To identify DNA methylation relevant to tumour behaviour, differences in DNA methylation patterns between groups were compared pairwise (Table 1). The number of statistically significant differentially methylated probes between groups in all comparisons is shown in Additional file 9: Table S3.

**Fig. 2:**
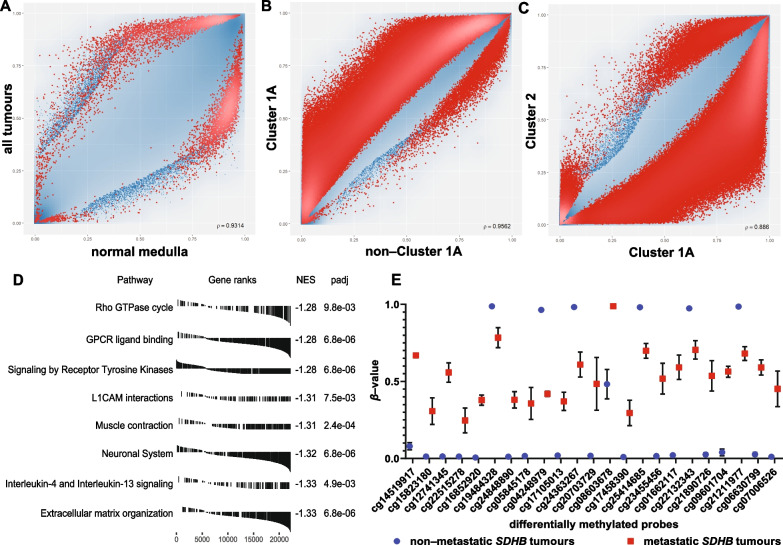
**A–C** Scatterplots for differentially methylated sites genome wide. Each point represents a probe of the 850 K array, and significantly differentially methylated probes (FDR < 0.05) are marked with red. Cluster 1A has the highest number of differentially methylated sites when compared to all other tumour samples (non-Cluster 1A) (**B**) or when compared only to Cluster 2 (**C**). **D** Top 10 gene set enrichment analysis (GSEA) results on differentially methylated promoters in Cluster 1A tumours after collapsing the redundant pathways with their corresponding gene-rank plots. Receptor tyrosine kinase and MAPK signalling pathways and the extracellular matrix are amongst the most affected. NES: normalised enrichment score. **F**: *β*-values of differentially methylated probes between metastatic and non-metastatic *SDHB* tumours. In the case of non-metastatic tumours, DMPs are in a binary state, mostly completely unmethylated with some completely methylated. In contrast, these loci in metastatic tumours have intermediate methylation levels. Mean ± standard deviation error bars

The highest number of differentially methylated probes was found in Cluster 1A compared to all other categories, consistent with the global DNA methylation analysis (Fig. 1), confirming that Cluster 2 tumours are more similar to normal tissue in terms of DNA methylation, than Cluster 1A. A total of 2938 probes were hypermethylated and 2992 hypomethylated in the comparison of all tumour samples compared to normal adrenal medulla (Fig. 2A). Cluster 1A exhibited 146,635 hypermethylated and 1539 hypomethylated probes compared to non-Cluster 1A tumours (Fig. 2B). A similar comparison with sporadic samples revealed 155,959 and 1474 differentially methylated sites, respectively. Differential methylation was of similar levels when all Cluster 1 tumours were aggregated (Cluster 1A + B) (Additional file 9: Table S3). These tumours share the pseudohypoxia phenotype but not the metabolic and biochemical aberrations of SDH deficiency. When compared to Cluster 2 tumours, Cluster 1A samples had 203,474 hypermethylated and 11,390 hypomethylated CpGs (Fig. 2C).

Tumours within Cluster 1A had fewer differentially methylated probes when compared to one another, than in inter-cluster pairwise comparisons (Additional file 9: Table S3). However, tumours within Cluster 2 (*NF1*, *RET* or *HRAS*) had no differentially methylated probes in any comparisons between this group. Sporadic tumours had fewer methylated probes than normal tissue (13,736 hypomethylated vs 4,737 hypermethylated), consistent with findings typical of DNA methylation states in cancer [15].

Compared to normal tissue derived from the adrenal medulla, phaeochromocytomas had few differences in DNA methylation with 3,355 hypermethylated and 713 hypomethylated CpG probes (Additional file 9: Table S3). Phaeochromocytomas were significantly different from head and neck paragangliomas with 1822 hypermethylated and 85,507 hypomethylated probes, and from extra-adrenal paragangliomas with 340 hypermethylated and 59,349 hypomethylated probes. These results could be partly explained by the higher frequency of Cluster 1A pathogenic variants in HNPGLs and EAPGLs compared to PCCs.

Pathway analyses discriminate PPGLs into groups according to genetic aetiology. Data derived from array-based DNA methylation analyses benefit from normalisation to account for multiple probes per gene set [62]. However, due to the large number of differentially methylated CpGs and their distribution, the results showed numerous significantly over-represented pathways. The use of more stringent selection criteria (FDR = 0.01 vs FDR = 0.05) on the differentially methylated probes used as input data did not alter this. The 20 most over-represented Reactome pathways (Benjamini–Hochberg adjusted *p* values 5.56 × 10^–23^–3.22 × 10^–6^) had ratios of 0.87 to 0.92, indicating that nearly all the genes associated with each pathway were affected (Additional file 3: Figure S3).

Most of the illuminated pathways in Cluster 1A tumours are associated with PPGLs and chromaffin biology, as expected, with neuronal system and synaptic transmission as central pathways. The extracellular matrix reorganisation pathway has been highlighted as a disrupted pathway in tumours with *SDHB* pathogenic variants and linked to tumour phenotypes such as EMT, cell invasion, migration and metastasis [72]. MAPK signalling, which is central to Cluster 2 tumorigenesis, was perturbed in Cluster 1A tumours via RTK signalling, MAPK signalling itself and downstream pathways involving Rho GTPases and G alpha (q) signalling. DNA methylation patterns at genes related to Wnt signalling were perturbed more broadly in this study [1].

High gene ratios across multiple pathways are not typical of over-representation results. Therefore, the approaches employed may have been influenced by the large number of differentially methylated CpGs altered in Cluster 1A tumours, so pre-ranked gene set enrichment analysis (GSEA), and a method used to add biological context was utilised [61, 73]. GSEA on differentially methylated CpGs in promoter regions of Cluster 1A tumours revealed very similar results to the over-representation analysis and included the terms extracellular matrix organisation, the L1 family of cell adhesion molecules (L1CAMs) interactions, signalling by RTK, VEGF signalling and Rho GTPase cycling (Fig. 2D).

Many differentially methylated promoters between phaeochromocytomas and head and neck paragangliomas belong to pathways related to development, including multiple homeobox genes, key transcriptional regulators (Additional file 10: Table S4). Of those, promoters of genes in the four *HOX* clusters had higher levels of DNA methylation in the phaeochromocytomas, whereas the other *HOX* genes were more methylated in head and neck paragangliomas (average Δ*β* = 0.29 and 0.2, respectively). Differentially methylated promoters in extra-adrenal paragangliomas also differed from phaeochromocytomas. Gene sets associated with the MAPK pathway were enriched in extra-adrenal PGLs, along with those related to extracellular matrix organisation, RTK signalling and the L1CAMs (Additional file 4: Figure S4). This deregulation of proteins related to the extracellular matrix could explain why extra-adrenal paragangliomas often show more aggressive tumour behaviour.

### DNA methylation patterns differ between metastatic and non-metastatic *SDHB* tumours

Tumours in patients harbouring mutations in *SDHB* have the highest risk of malignancy. However, so far, there are no specific prognostic factors or biomarkers to identify tumours at high likelihood for becoming malignant that have been clinically utilised. Seeing that hypermethylation is a central factor to these tumours, DNA methylation in the subset of *SDHB* tumours for which clinical data were available (*n* = 8) were examined.

Differentially methylated loci in the three *SDHB* patients (37.5%) who developed distant metastatic events after primary tumour formation compared to the other *SDHB* patients were examined (Additional file 11: Table S5). Twenty-three probes were differentially methylated (Fig. 2F). The majority of non-metastatic samples were either unmethylated or completely methylated (values of 0 or 1) with extremely low inter-sample variance. Metastatic tumours on the other hand, appear to be in an intermediate methylation state at these loci, on average. This pattern illustrates a distinction in DNA methylation state that follows a defined phenotype, suggesting loci of interest for further study.

One differentially methylated probe (cg19484328) is in the gene body of *EPHA4* that codes for EPH Receptor A4, an RTK of the ephrin family, and another (cg12741345) is in the gene body of *EFNA3*, an ephrin which is a ligand of EPHA4. Ephrin activation modulates cell morphology and in integrin-dependent cell adhesion [74]. Tissue hypoxia has been shown to regulate *EFNA3* [75] that is a clinical prognostic and therapeutic predictor in lung adenocarcinoma and hepatocellular carcinoma [76, 77].

### Histone post-translational modification patterns distinguish cluster 1A PPGL tumours from other classes

**Fig. 3:**
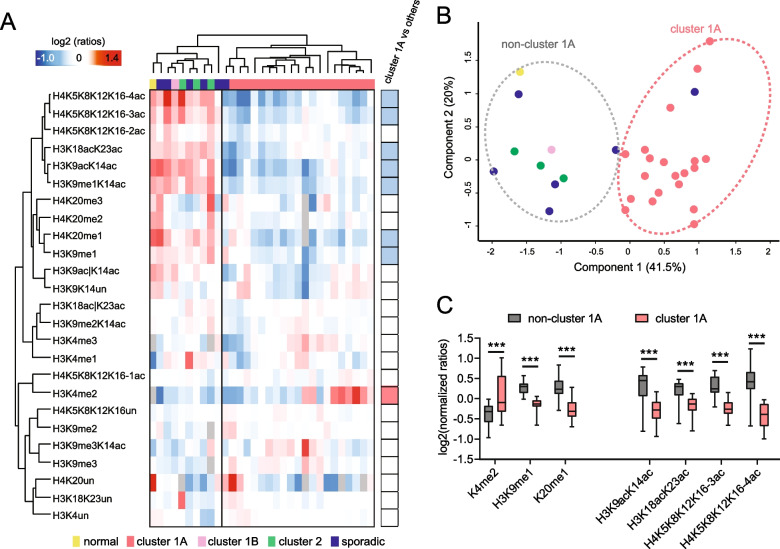
**A** Heatmap display of histone PTM levels in PPGL patient tissues. L/H (light/heavy) relative abundances ratios were obtained using a spike-in strategy (light channel: sample, heavy channel: spike-in standard) and were normalised over the average ratios across samples. The grey colour indicates peptides that were not quantified. Right panel: Modified peptides were compared in samples belonging to Cluster 1A or other tumour classes by multiple *t* test. The red colour indicates a significant increase (FDR < 0.05) in Cluster 1A, and the blue colour indicates a significant decrease. **B** PCA analysis based on quantitative histone PTM data obtained from the samples shown in A. **C** Boxplot display of the data shown in A, for selected PTMs. *** FDR < 0.001 by multiple unpaired *t* test

Histone PTM levels in 31 patient samples were examined, including one normal tissue, 20 Cluster 1A tumours and 10 tumours in other classes (Fig. 3; Table 1). Quantitative mass spectrometry analysis quantified 25 differentially modified histone H3 and histone H4 peptides to reveal differences between Cluster 1A tumours and other tumours, which separate into two well-defined clusters by both unsupervised hierarchical clustering and PCA analysis (Fig. 3A, B). The normal tissue, Cluster 2 and sporadic tumours clustered together but separately from Cluster 1A tumours. The significant changes included a general decrease in hyper-acetylated peptides (the tri- and tetra-acetylated form of the histone H4 tail, and the di-acetylated form of histone H3 9–17 and 18–26 peptides), of H3K9me1 and H4K20me1, and an increase in H3K4me2 in Cluster 1A tumours (Fig. 3C). The decrease in overall histone acetylation fits with the observed increase in DNA methylation, as both are markers of silenced chromatin.

### Gene expression comparison to differential DNA methylation in PPGL tumours

To identify the differences between tumours with different underlying pathogenic variants, PCA on normalised gene counts was performed (Additional file 5: Figure S5). Based on their transcriptomic profiles, PPGL samples from Cluster 1A form a group that is distinct from sporadic samples, in concordance with DNA methylation. *VHL* samples are more similar in their expression profile to sporadic rather than Cluster 1A tumours indicating a high degree of similarity after dimensionality reduction.

**Fig. 4:**
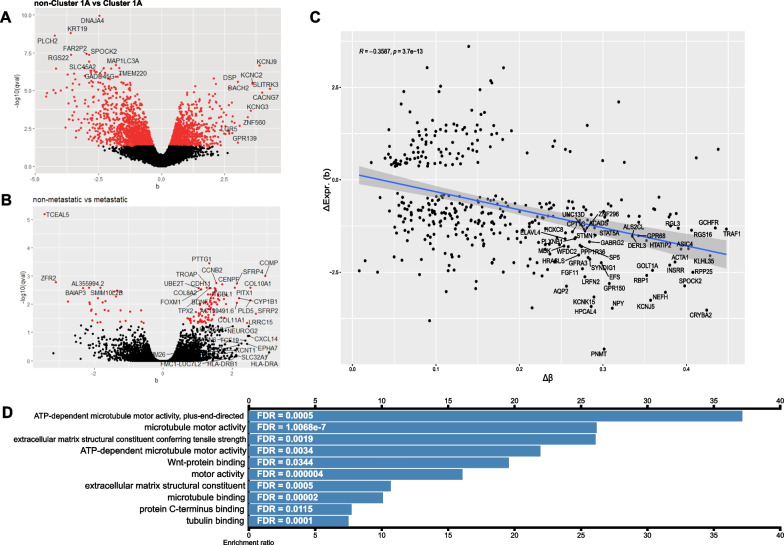
**A–B** Volcano plots showing the effect size (b) of differential gene expression and its statistical significance for the comparisons Cluster 1A versus non-Cluster 1A and metastatic versus non-metastatic tumours from the RNA-seq data. **C** Correlation plot of differentially methylated promoters and differentially expressed genes in metastatic tumours shows an inverse relationship between the more differentially methylated (higher Δ*β*) and the level of downregulation. Pearson’s correlation coefficient *ρ* = −0.3587, *p* = 3.7 × 10^–13^. **D** Over-representation analysis of upregulated differentially expressed genes between metastatic and non-metastatic tumours for molecular function ontology revealed enriched terms were mainly related to motor proteins, the *Wnt*-pathway and the extracellular matrix

Gene expression levels from Cluster 1A and non-Cluster 1A tumour samples were compared with one another (Fig. 4A). In addition to the previously described genes *KRT19*, *SPOCK2* and *DNAJA4* which are downregulated in *SDHx* tumours [9], the tumour suppressor *RGS22* implicated in EMT and metastasis is differentially expressed [78, 79]. Downregulation of these genes may be involved in the invasive phenotype of SDH-deficient cells.

Data from non-metastatic versus metastatic tumours were compared to detect differences and identify genes involved in metastatic tumour behaviour (Fig. 4B). Some of the most upregulated genes in the metastatic tumours are the pituitary tumour-transforming gene (*PTTG1*), a marker of invasion in many tumour types including endocrine cancers [80] and the pro-proliferative cyclin B2 *CCNB2*. ECM-related integrin, beta-like 1 (*ITGBL1*), cadherin 11 (*CDH11*) and trophinin-associated protein (*TROAP*) were upregulated, perhaps reflecting a reorganisation of the tumour microenvironment that is permissive of invasion and metastasis.

The effect sizes (*b* statistic) of differentially expressed genes between metastatic and non-metastatic tumours were compared to the DNA methylation levels of their respective promoters (Fig. 4C). A moderate anticorrelation (Pearson’s *ρ* = −0.3587, *p* = 3.7 × 10^–13^) between levels of differential promoter DNA methylation and differential gene expression was observed. A similar anticorrelation was observed comparing sporadic to *SDHB* tumours (Pearson’s *ρ* = −0.3515, *p* = 2.7 × 10^–14^) but not between *VHL* and *SDHB* tumours (Pearson’s *ρ* = −0.1409, *p* = 0.32) (Additional file 6: Figure S6).

### Pathway analysis

GSEA enrichment analyses comparing non-metastatic versus metastatic tumours were performed. The two most enriched gene sets were “regulation of cell division” (GO:0051302) with normalised enrichment score of 2.9 (FDR < 1 × 10^–6^) and “tricarboxylic acid metabolic process” (GO:0072350) which had a negative normalised enrichment score of −2.74 (FDR < 0.01), indicating downregulation of the genes in this dataset (Additional file 12: Table S6).

Over-representation analysis (ORA) for Biological Process and Cellular Component gene ontology terms and Reactome pathway analysis on upregulated genes (Additional file 13: Table S7, Additional file 14: Table S8) and downregulated genes (Additional file 15: Table S9, Additional file 16: Table S10, Additional file 17: Table S11) in Cluster 1A compared to non-Cluster 1A tumours highlighted perturbed pathways such as extra cellular regulation of signal transduction and regulation of transcription.

ORA on genes upregulated in metastatic tumours compared to non-metastatic ones revealed perturbations in genes associated with the extracellular matrix, cell motility and replication regulation (Additional file 18: Table S12, Additional file 19: Table S13, Additional file 20: Table S14).

ORA on the Pharmacogenomics Knowledgebase (PharmGKB) that curates data on the impact of human genetic variation on drug responses was interrogated using the upregulated genes in the metastatic tumours as input. The results pointed to the drug group taxanes, the commonly used chemotherapy agents docetaxel, epirubicin and paclitaxel (Additional file 21: Table S15), as likely to yield a favourable/mechanistically relevant response.

Finally, we analysed differential expression between metastatic sporadic and metastatic *SDHx* tumours (all of which were *SDHB* mutated). The only significantly upregulated pathway in *SDHB* tumours was the TCA cycle and respiratory electron transport, highlighting the metabolic dysregulation in these tumours.

## Discussion

PPGLs present with a diverse group of phenotypes; although the majority of these tumours remain localised, metastatic events are not uncommon and currently it is not possible to predict in which patients they will occur, or when. Tumours with an *SDHB* pathogenic variant confer the highest risk of metastasis. It is not well understood why pathogenic variants in the *SDHB* subunit gene are more likely to result in metastatic tumours than in other SDH subunits or other PPGL genes. A plethora of potential mechanisms responsible for this have been suggested, including activation of epithelial-to-mesenchymal transition pathways, reactive oxygen species imbalance, metabolic rewiring, angiogenesis, pseudohypoxia and iron homeostasis [72, 81–83].

*SDHB* PPGLs also exhibit profound hypermethylator phenotype which we characterised at high resolution in this study. Whether this extreme hypermethylation results in gene expression alterations that are directly responsible for the more aggressive phenotype, or the consequences of hypermethylation drive the emergence of genetically heterogeneous subclones of cancer cells is not known. Using mass spectrometry, we also studied histone PTMs for the first time in PPGLs, finding that *SDHx* tumours have a significant decrease in hyper-acetylated marks. Since there are no curative therapies for metastatic disease, which often presents many years later, studying these tumours at the transcriptomic and epigenomic level can inform our understanding of the mechanistic processes that underpin them.

Advances in omics technologies have improved the understanding of tumour cell development and determinants of progression to metastatic states [84]. Despite the accumulated data on tumours associated with known pathogenic variants, the ability to use this information in predictive diagnostic test development for clinical applications remains limited.

A recent large-scale genomic profiling study of 156 PPGLs, with a range of genetic aetiologies, from 128 unrelated patients, found that although PPGL tumour formation is driven by germline or somatic mutations, the process of metastasis can involve immunologic and cell-extrinsic events. In addition to genetic features associated with metastasis such as high mutational load, microsatellite instability, somatic copy number alteration burden associated with ATRX/TERT alterations, or *CDK1* overexpression and the presence of MAML3-fusions as potential markers, the study also found that immunogenic markers, which while heterogeneous, did point to the potential for immunologic characterisation to be considered in the clinical management of PPGL prognostication [85].

A machine learning-based tool based on clinical phenotypes has also shown promise in predicting metastatic disease [86]. This type of approach could also have utility in the prognostication of metastatic potential in PPGLs. DNA methylation and histone PTM data can be input to these models, potentially increasing their sensitivity and accuracy. This offers the potential of a better understanding of the mechanisms of tumour development and molecular targets leading to the development of triage tools with immediate clinical application. Currently, the utility for routine clinical practice of the known molecular markers is unclear because these types of features are not usually available pre-operatively, so more development is needed.

Here, hypermethylation of the genome in PPGL tumours has been substantiated and characterised in high resolution. As expected, Cluster 1A tumours have higher DNA methylation levels with more DNA methylation changes as compared to normal tissue than either sporadic or Cluster 2 tumours. This hypermethylation may mediate the unfavourable tumour behaviour through the silencing of promoter(s) of tumour suppressor or other genes that normally suppress metastatic tendency. This type of DNA methylation assay examines DNA methylation agnostic to sequence or as annotated functional elements, and in both approaches, *SDHB* pathogenic variants stand out as the most hypermethylated tumour types. Non-metastatic tumours had either completely DNA methylated or unmethylated sites, with low inter-sample variance. Metastatic tumours on the other hand consistently showed an intermediate DNA methylation state.

Pathway analysis comparing aggressive and non-aggressive tumours highlighted pathways and genes that may contribute to aggressive tumour behaviour. Genes highlighted include the proto-oncogene *PTTG1* found to be significantly upregulated in metastatic tumours, consistent with studies that have associated it with metastatic behaviour of endocrine and non-endocrine tumours [80]. It is a potential candidate marker of aggressiveness that, either alone or as part of scoring systems, could stratify patients according to their risk for metastasis. Downregulation of the tumour suppressor gene *RGS22* suggests a potential involvement in the invasive phenotype of *SDH*-deficient cells.

DNA methylation is highly tissue-specific both during development and in differentiated tissues. The location and/or tumour cell of origin therefore could be a factor that contributes to the pattern of DNA methylation. For PPGL, the site of tumour origin is associated with differences in clinical outcome, with extra-adrenal paragangliomas generally being more aggressive than phaeochromocytomas and head and neck paragangliomas. To note, the DNA methylation signatures of extra-adrenal paragangliomas are different not only from that of normal adrenal medulla, but also from phaeochromocytomas, despite their developmental and anatomical proximity. These differences in methylation patterns could potentially relate to the differences in clinical phenotype.

Developmental genes, including multiple *HOX* genes, had differentially methylated promoters between tumour subtypes. Deregulation of promoter methylation of *HOX* genes has been observed in numerous cancers [87] and is implicated in cancer progression through the induction of proliferation, angiogenesis, as well as cell invasion, adhesion and migration.

This study employed a proteomics approach using mass spectrometry to investigate histone modification in PPGLs. The 25 histone modifications characterised globally, similarly to DNA methylation, readily distinguished PPGL Cluster 1A tumours from other pathogenic variants. Specifically, an increase in H3K4me2 and decrease in hyper-acetylated peptides were hallmarks of Cluster1A. Although these studies provide an insight into PPGL tumours and their epigenomes across a range of pathogenic variants, they did not highlight a single biomarker for tumour behaviour. However, various histone deacetylase inhibitors have shown promising results in subsets of patients with PPGLs or other neuroendocrine tumours [88]. Based on the heterogeneity of acetylated histone marks in these tumours, with Cluster 1A having significantly lower levels, which could be an epigenetic vulnerability that can be targeted by histone deacetylase inhibitors.

PPGLs have been classified by their transcriptomes into three, well-established, groups. Herein, we examined RNA-seq data from different PPGLs and detected both known differentially expressed genes that support Cluster 1A as distinct in terms of gene expression, as well as novel genes, including *PTTG1* and *RGS22,* that are differentially expressed. Pathway analyses highlighted, the TCA cycle, and a number of disease-relevant themes emerged including extracellular matrix organisation and RTK signalling which might explain the aggressive clinical phenotypes that these tumours can exhibit.

Recent advances in single-cell RNA sequencing could provide a way to profile individual tumour cells and small groups of cells to detect differences between metastatic and non-metastatic tumours. Identifying potential rare cell populations with a “pre-metastatic”-like expression profile/state that bulk-sequencing approaches may not be able to detect, could reveal more about the genes and mechanisms behind these phenotypic differences.

## Conclusions

This report substantiates transcriptomic and epigenomic distinctions between PPGL tumours with pathogenic variants in different genes. We used a multi-omic approach of transcriptomic and epigenomic data to identify biologically relevant genes that distinguish Cluster 1A tumours and identified DNA methylation differences that could account for the metastatic behaviour we more often see in *SDHB*-related PPGLs.

### Supplementary Information

**Additional file 1**. **Figure S1**. Flow chart detailing the origin of the human tissue samples used the study.

**Additional file 2**.  **Figure S2**. Principal component analysis (PCA) of the 50000 most variable probes in the DNA methylation analysis of Cluster 1B and sporadic versus Cluster 2 tumours, revealing that the DNA methylation variance is driven by the underlying pathogenic variant.

**Additional file 3**. **Figure S3**. Overrepresentation pathway analysis of DNA methylation data for Cluster 1A versus non-Cluster 1A tumours.

**Additional file 4**. **Figure S4**. Gene set enrichment analysis for differentially methylated promoters for EAPGLs versus PCCs.

**Additional file 5**. **Figure S5**. Principal component analysis on normalised gene counts was performed on the RNA-seq data for 29 tumour samples. PPGL samples from Cluster 1A form a group that is distinct from sporadic samples, in concordance with DNA methylation.

**Additional file 6**. **Figure S6**. Relationship between promoter DNA methylation differences and changes in the corresponding gene expression in *VHL* versus *SDHB* samples did not reveal statistically significant correlation. 

**Additional file 7**. **Table S1**. List of all samples, their mutation, tumour location, phenotype, data generated and sample source.

**Additional file 8**.  **Table S2**. Mass Spec analysis of histone post translational modifications detailing the log_2_ (L/H ratios) for all the samples analysed.

**Additional file 9**.  **Table S3**. Differential methylation summary table. The number of statistically significant differentially methylated probes between groups in all comparisons is shown.

**Additional file 10**.  **Table S4**. Differentially methylated loci in the three SDHB patients (37.5%) who developed distant metastatic events after primary tumour formation compared to the other SDHB patients were examined. Location of promoters of genes in EAPGL versus PCC are included.

**Additional file 11**. **Table S5**. Differentially methylated loci in the three *SDHB* patients who developed distant metastatic events after primary tumour formation compared to the other *SDHB* samples.

**Additional file 12**.  **Table S6**. GSEA enrichment analyses comparing non-metastatic versus metastatic tumours.

**Additional file 13**.  **Table S7**. Over-representation analysis (ORA) on upregulated genes in Cluster 1A compared to non-Cluster 1A tumours for Biological Process and Cellular Component gene ontology terms. Perturbed biological processes included “negative regulation of execution phase of apoptosis” (GO:1900118) with enrichment ratio (ER) 27.43 and FDR < 0.001, “extracellular regulation of signal transduction” (GO:1900115, ER=16.625, FDR < 0.001) and “regulation of transcription involved in cell fate commitment” (GO:0060850, ER=16.136, FDR=0.01).

**Additional file 14**.  **Table S8**. Reactome pathway analysis on upregulated genes in Cluster 1A compared to non-Cluster 1A tumours. From the Reactome Knowledgebase, the pathway related to RTK-protein phosphatases (R-HSA-388844) had ER=17.947 and FDR < 0.01.

**Additional file 15**.  **Table S9**. Over-representation analysis (ORA) on downregulated genes in Cluster 1A compared to non-Cluster 1A tumours for Biological Process and Cellular Component gene ontology terms. Affected biological processes included inner dynein arm assembly (GO:0036159, ER=12.171, FDR=0.02), positive regulation of protein processing (GO:0010954, ER=7.86, FDR=0.03) and positive regulation of lipid biosynthetic process (GO:0046889, ER=4.4839, FDR=0.03). Some of the cellular components involved were collagen-containing extracellular matrix (GO:0062023, ER=3.4064, FDR < 0.001) and the neuronal cell body (GO:0043025, ER=3.1354, FDR=9.32 x 10-7).

**Additional file 16**.  **Table S10**. Reactome pathway analysis revealed multiple pathways were perturbed including those related to epigenetic regulation: “passive transport by aquaporins” (R-HSA-432047, ER=13.712, FDR < 0.01), “RNA polymerase I promoter opening” (R-HSA-73728, ER=5.65, FDR < 0.001), “DNA methylation” (R-HSA-5334118, ER=5.4849, FDR=0.004) and “packaging of telomere ends” (R-HSA-171306, ER=5.4849, FDR < 0.01).

**Additional file 17**.  **Table S11**. Reactome pathway analysis on downregulated genes in Cluster 1A compared to non-Cluster 1A tumours for Biological Process and Cellular Component gene ontology terms.

**Additional file 18**.  **Table S12**. Some gene ontology sets from the Biological Process analysis include “positive regulation of DNA topoisomerase (ATP-hydrolyzing) activity” (GO:2000373, ER=71.214, FDR=0.02), “regulation of exit from mitosis” (GO:0007096, ER=35.607, FDR < 0.001) and “regulation of mitotic sister chromatid separation” (GO:0010965, ER=26.705, FDR < 0.001).

**Additional file 19**.  **Table S13**. In Molecular Function gene sets (Figure 4D) “ATP-dependent microtubule motor activity, plus-end-directed” (GO:0008574, ER=37.164, FDR < 0.001), “microtubule motor activity” (GO:0003777, ER=26.194, FDR=1 x 10-7) and “extracellular matrix structural constituent conferring tensile strength” (GO:0030020, ER=26.115, FDR < 0.01) were the most enriched.

**Additional file 20**.  **Table S14**. The Reactome pathways “G2/M DNA replication checkpoint” (R-HSA-69478, ER=72.779, FDR=0.015), “condensation of prometaphase chromosomes” (R-HSA-2514853, ER=66.163, FDR < 0.001), “kinesins” (R-HSA-983189, ER 25.537, FDR=8.35 x 10-8) and “amplification of signal from the kinetochores” (R-HSA-141424, ER=18.953, FDR=1.38 x 10-8) were enriched.

**Additional file 21**.  **Table S15**. ORA on the Pharmacogenomics Knowledgebase (PharmGKB) was interrogated using the upregulated genes in the metastatic tumours as input. The results pointed to the drug group taxanes (PA150481189, ER=21.722, FDR=3.75 x 10-10) docetaxel (PA449383, ER=19.795, FDR < 0.001), epirubicin (PA449476, ER=14.405, FDR < 0.01) and paclitaxel (PA450761, ER=13.986, FDR < 0.001).

## Data Availability

The DNA methylation raw data have been deposited into ArrayExpress with accession number E-MTAB-13433. The mass spectrometry proteomics data have been deposited to the ProteomeXchange Consortium via the PRIDE partner repository with dataset identifier PXD025689. The RNA-seq data not already in the public domain will be deposited into dbGAP/EGA and can be requested from KLN.
