# Supplementary figures and images for: A comprehensive characterisation of phaeochromocytoma and paraganglioma tumours through histone protein profiling, DNA methylation and transcriptomic analysis genome wide
Source: Clin Epigenetics. 2023 Dec 20;15:196. doi: 10.1186/s13148-023-01598-3 (PMC10734084; doi:10.1186/s13148-023-01598-3)

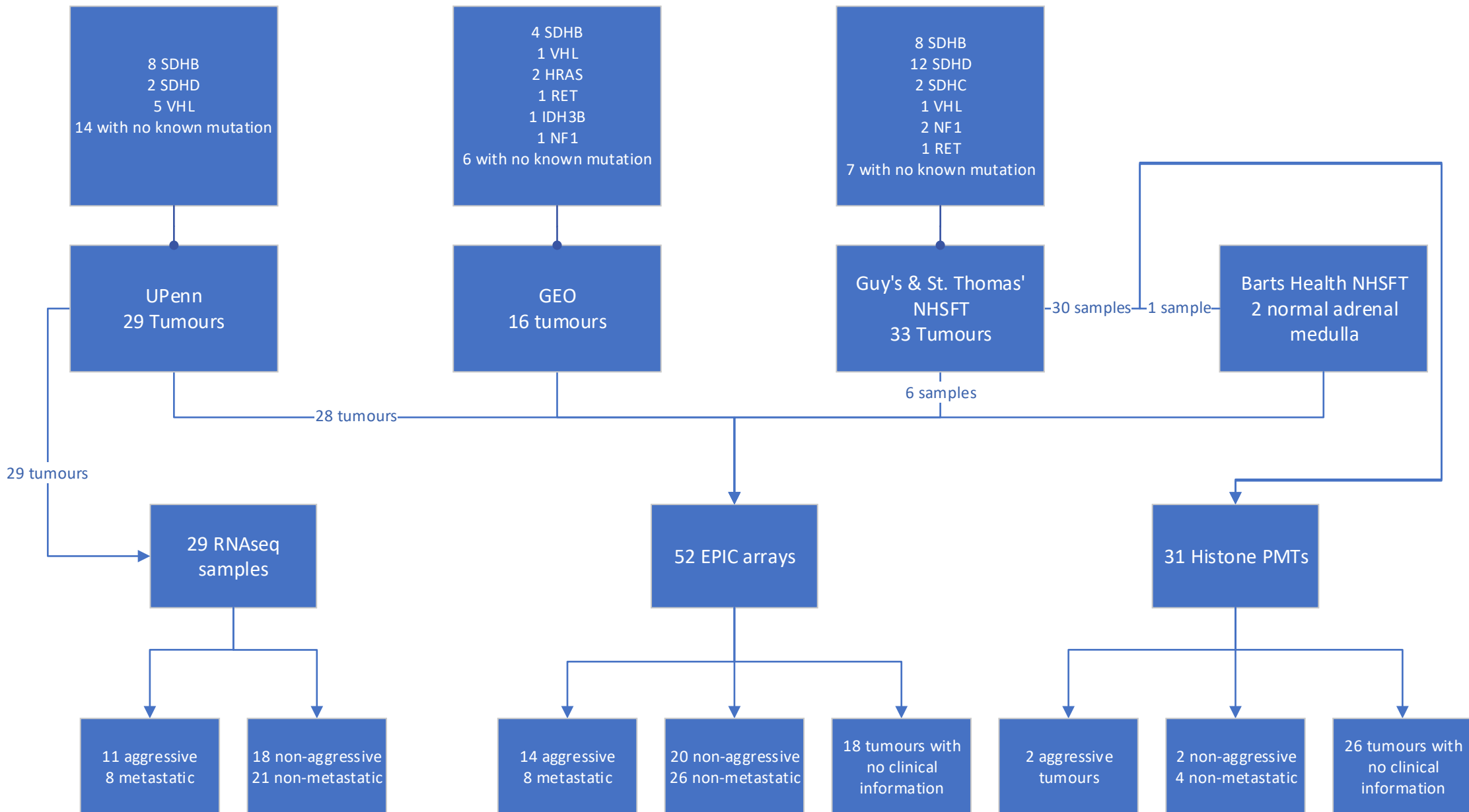

Supplement: Supplementary file 1 — Additional file 1. Figure S1. Flow chart detailing the origin of the human tissue samples used the study. [file 13148_2023_1598_MOESM1_ESM.pdf]

PCA of the 50,000 most variable probes

cluster 1 cluster 1b cluster 2 normal sporadic

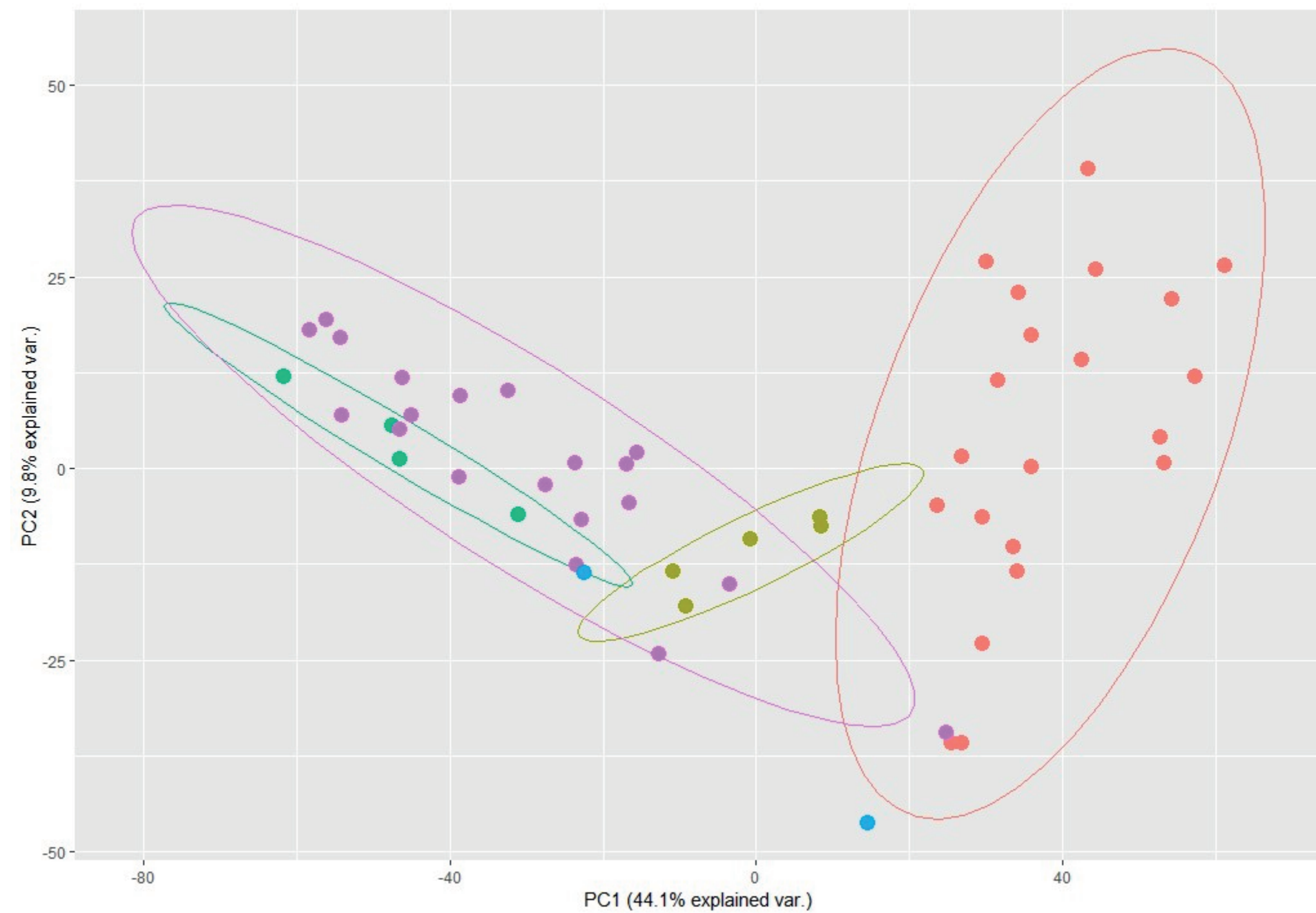

Supplement: Supplementary file 2 — Additional file 2. Figure S2. Principal component analysis (PCA) of the 50000 most variable probes in the DNA methylation analysis of Cluster 1B and sporadic versus Cluster 2 tumours, revealing that the DNA methylation variance is driven by the underlying pathogenic variant. [file 13148_2023_1598_MOESM2_ESM.pdf]

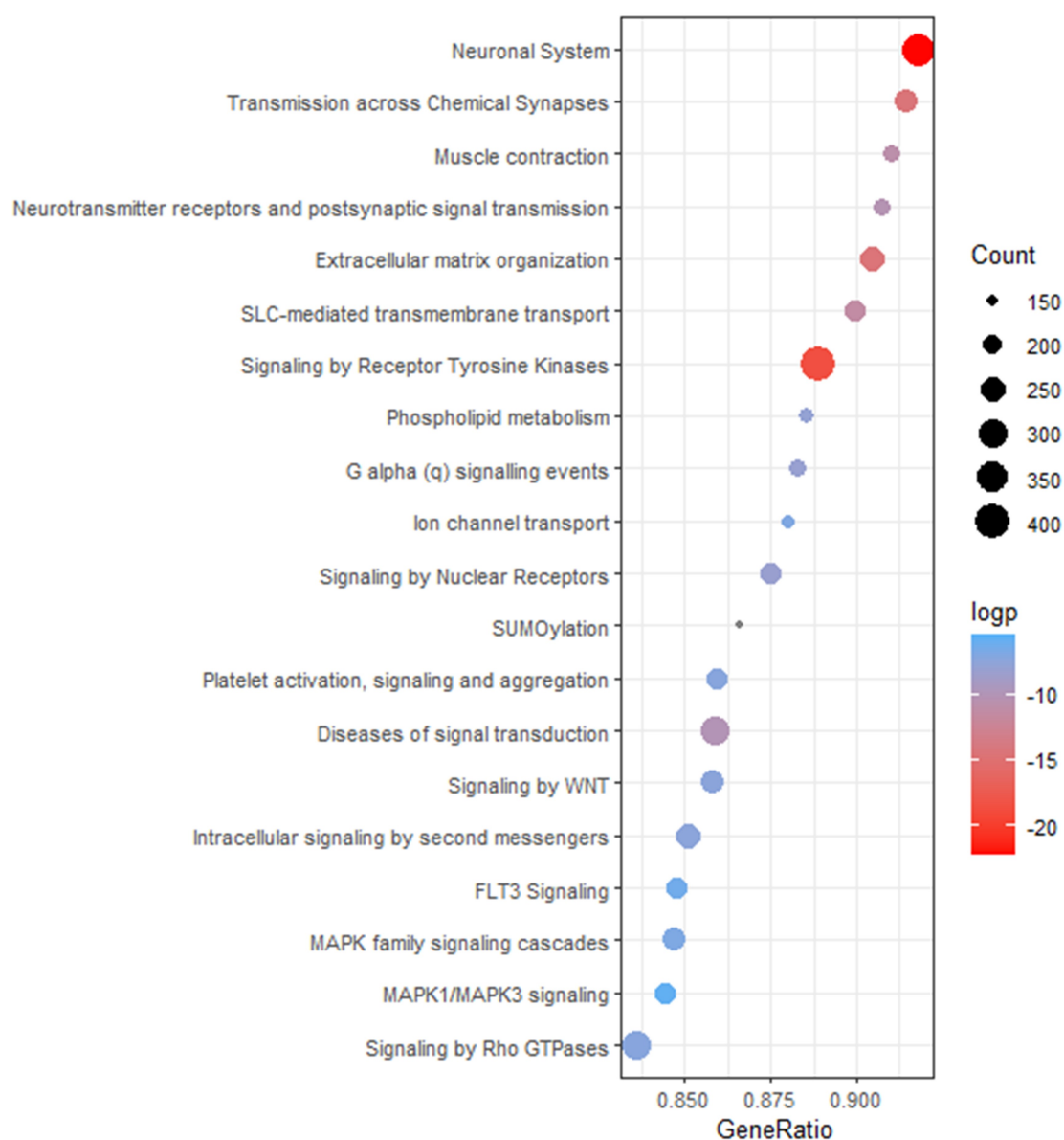

Supplement: Supplementary file 3 — Additional file 3. Figure S3. Overrepresentation pathway analysis of DNA methylation data for Cluster 1A versus non-Cluster 1A tumours. [file 13148_2023_1598_MOESM3_ESM.pdf]

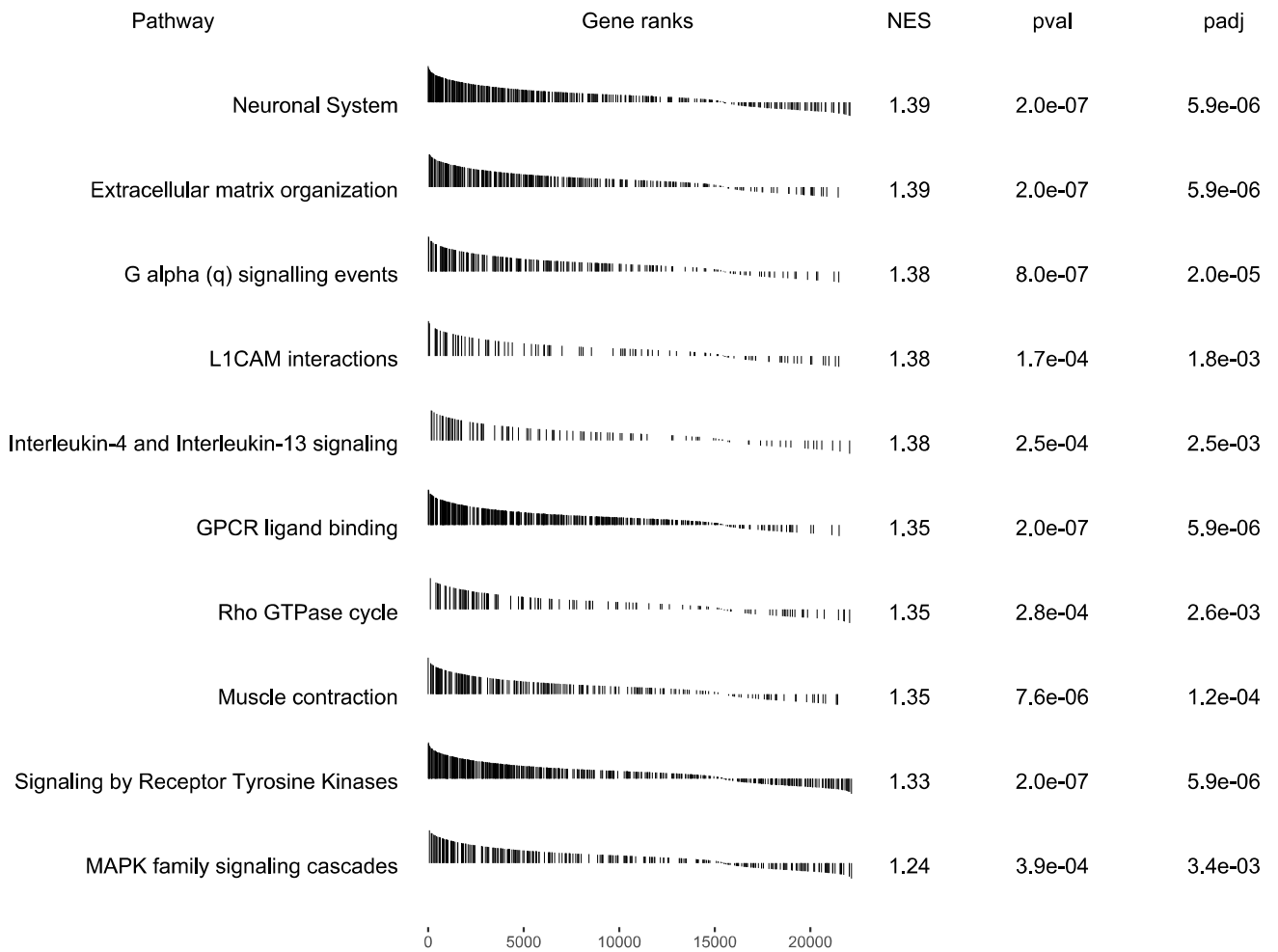

Supplement: Supplementary file 4 — Additional file 4. Figure S4. Gene set enrichment analysis for differentially methylated promoters for EAPGLs versus PCCs. [file 13148_2023_1598_MOESM4_ESM.pdf]

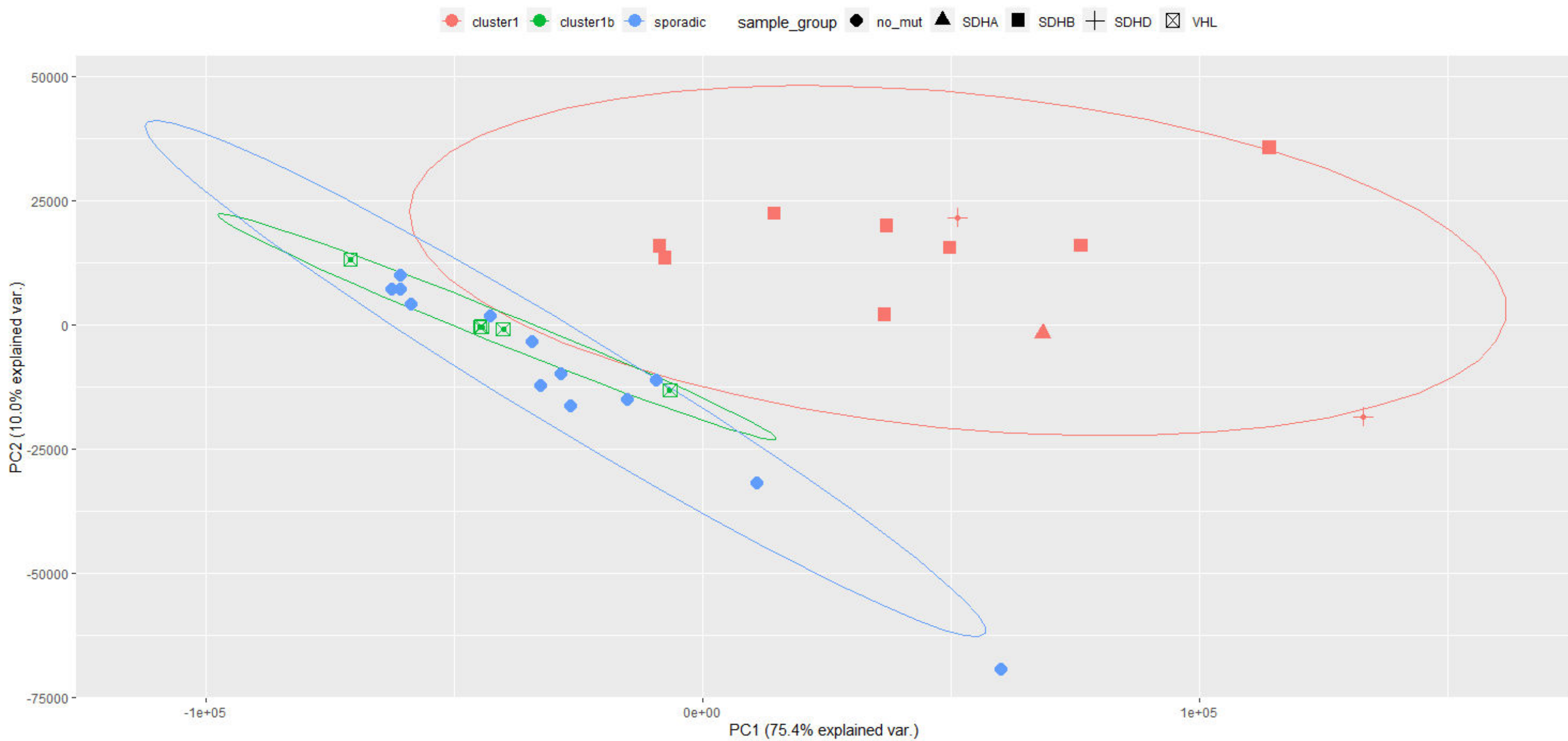

Supplement: Supplementary file 5 — Additional file 5. Figure S5. Principal component analysis on normalised gene counts was performed on the RNA-seq data for 29 tumour samples. PPGL samples from Cluster 1A form a group that is distinct from sporadic samples, in concordance with DNA methylation. [file 13148_2023_1598_MOESM5_ESM.pdf]

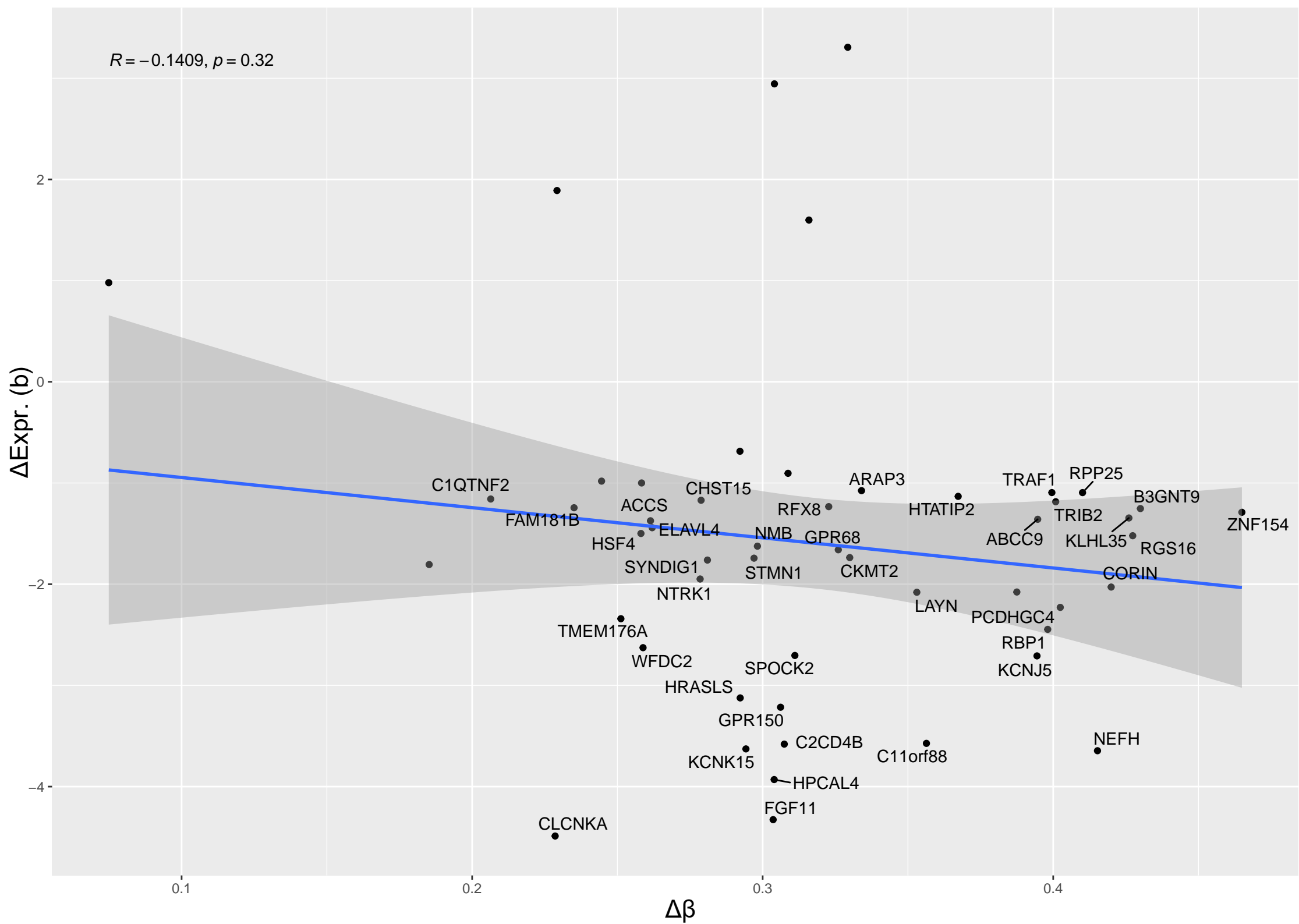

Supplement: Supplementary file 6 — Additional file 6. Figure S6. Relationship between promoter DNA methylation differences and changes in the corresponding gene expression in VHL versus SDHB samples did not reveal statistically significant correlation. [file 13148_2023_1598_MOESM6_ESM.pdf]
